# *Lacticaseibacillus casei* Strain T21 Attenuates *Clostridioides difficile* Infection in a Murine Model Through Reduction of Inflammation and Gut Dysbiosis With Decreased Toxin Lethality and Enhanced Mucin Production

**DOI:** 10.3389/fmicb.2021.745299

**Published:** 2021-12-01

**Authors:** Wimonrat Panpetch, Pornpimol Phuengmaung, Thanya Cheibchalard, Naraporn Somboonna, Asada Leelahavanichkul, Somying Tumwasorn

**Affiliations:** ^1^Department of Microbiology, Faculty of Medicine, Chulalongkorn University, Bangkok, Thailand; ^2^Department of Microbiology, Faculty of Science, Chulalongkorn University, Bangkok, Thailand; ^3^Microbiome Research Unit for Probiotics in Food and Cosmetics, Chulalongkorn University, Bangkok, Thailand; ^4^Department of Microbiology, Faculty of Medicine, Center of Excellence in Immunology and Immune-Mediated Diseases, Bangkok, Thailand

**Keywords:** *Lacticaseibacillus casei* T21, probiotics, proinflammatory cytokines, *Clostridioides difficile*, gut dysbiosis, inflammation, toxin lethality

## Abstract

*Clostridioides difficile* is a major cause of diarrhea in patients with antibiotic administration. *Lacticaseibacillus casei* T21, isolated from a human gastric biopsy, was tested in a murine *C. difficile* infection (CDI) model and colonic epithelial cells (Caco-2 and HT-29). Daily administration of *L. casei* T21 [1 × 10^8^ colony forming units (CFU)/dose] for 4 days starting at 1 day before *C. difficile* challenge attenuated CDI as demonstrated by a reduction in mortality rate, weight loss, diarrhea, gut leakage, gut dysbiosis, intestinal pathology changes, and levels of pro-inflammatory cytokines [interleukin (IL)-1β, tumor necrosis factor (TNF)-α, macrophage inflammatory protein 2 (MIP-2), and keratinocyte chemoattractant (KC)] in the intestinal tissue and serum. Conditioned media from *L. casei* T21 exerted biological activities that fight against *C. difficile* as demonstrated in colonic epithelial cells by the following: (i) suppression of gene expression and production of IL-8, an important chemokine involved in *C. difficile* pathogenesis, (ii) reduction in the expression of *SLC11A1* (solute carrier family 11 member 1) and *HuR* (human antigen R), important genes for the lethality of *C. difficile* toxin B, (iii) augmentation of intestinal integrity, and (iv) up-regulation of *MUC2*, a mucosal protective gene. These results supported the therapeutic potential of *L. casei* T21 for CDI and the need for further study on the intervention capabilities of CDI.

## Introduction

*Clostridioides difficile*, an anaerobic Gram-positive spore-forming bacillus (Kachrimanidou and Malisiovas, 2011), is one of the important causative organisms of diarrhea in hospitalized patients who receive antibiotics (Kelly et al., 1994b; Bartlett, 2002; Aslam et al., 2005). Clinical symptoms of *C. difficile* infection (CDI) vary from mild diarrhea (usually self-limited) to pseudomembranous colitis with severe sepsis (Mylonakis et al., 2001) and/or toxic megacolon (Kuehne et al., 2011). The pathogenesis of CDI is associated with antibiotic-induced gut dysbiosis that facilitates *C. difficile* colonization and toxin production (Mooyottu et al., 2017). Two protein exotoxins referred to as toxin A (TcdA) and toxin B (TcdB) are the major virulence factors contributing to CDI (Lyerly et al., 1988; Voth and Ballard, 2005; Kuehne et al., 2011). Binding of TcdA and TcdB to specific receptors on the surface of intestinal epithelial cells stimulates the secretion of several pro-inflammatory cytokines and chemokines (Hodges and Gill, 2010). Both toxins cause the loss of intestinal epithelial barrier function (gut leakage) by glucosylating Rho GTPases, which causes actin cytoskeleton rearrangement, tight junction disruption, and enterocyte cell death (Pothoulakis, 2000; Aktories and Barbieri, 2005; Jank and Aktories, 2008; Kuehne et al., 2011; Chen et al., 2015). In addition, binary toxin (*C. difficile* transferase, CDT) is observed in some *C. difficile* strains that cause severe CDI. This toxin is an ADP-ribosyltransferase that causes depolymerization of F-actin and rearrangement of the actin cytoskeleton (Gerding et al., 2014; Aktories et al., 2018).

The pathogenic effects of TcdA and TcdB have been studied extensively. Epithelial cells demonstrate a decrease in transepithelial electrical resistance (TEER) and an increase in paracellular permeability after toxin activation, indicating that *C. difficile* toxins disrupt gut tight junctions (Hecht et al., 1988; Nusrat et al., 2001; Zemljic et al., 2010). Toxin-activated intestinal epithelial chemotactic mediators, such as interleukin (IL)-8, cause an accumulation of neutrophils and lymphocytes (inflammatory colitis) and other clinical signs of infectious diarrhea (such as white blood cell in feces) (Viswanathan et al., 2009; Sun et al., 2010). CDI not only causes local intestinal inflammation but also induces systemic inflammation from gut leakage-induced bacteremia. Disruption of gut tight junctions also allows for the transfer of intestinal contents, including TcdA and TcdB from *C. difficile*, into the circulation, resulting in the activation of various immune cells in the bloodstream (Viswanathan et al., 2009; Sun et al., 2010). Subsequently, the activated immune cells systemically secrete several pro-inflammatory cytokines, which lead to systemic inflammatory responses and sepsis. In addition to bacterial factors, host factors also contribute to the severity of CDI. Notably, a previous report identified solute carrier family 11 member 1 gene (*SLC11A1*), which enhances TcdB lethality by the increased Rho GTPase glucosylation, and the suppression of *SLC11A1* resulted in reduced toxin sensitivity. In addition, the up-regulation of *SLC11A1* requires the RNA-binding protein HuR or human antigen R (encoded in *HuR*) to stabilize the mRNA (Feng and Cohen, 2013).

Probiotics are live microorganisms that, when administered in adequate amounts, confer a health benefit on the host (FAO and WHO, 2001; Hill et al., 2014). Probiotics have been a popular approach for the prevention and improvement of treatment efficacy of human diseases. Several meta-analyses suggest that probiotics, mainly *Lactobacillus*, are effective for preventing *C. difficile*-associated diarrhea (Ritchie and Romanuk, 2012; Goldenberg et al., 2017; Shen et al., 2017). It has been suggested that probiotic administration counteracts gut dysbiosis caused by antibiotics or infections (Reid et al., 2011), resulting in the restoration of gut microbiota diversity, which plays a crucial role in the prevention of CDI (Kachrimanidou and Tsintarakis, 2020). Specific strains of *Lactobacillus* spp. effectively inhibit the pathogenicity of *C. difficile* both *in vitro* (Banerjee et al., 2009; Trejo et al., 2010; Spinler et al., 2017) and *in vivo* (Leelahavanichkul et al., 2016) and secrete several anti-inflammatory substances that attenuate enterocyte injury from several insults (Boonma et al., 2014; Panpetch et al., 2016, 2018).

Despite a variety of probiotics, we speculated that indigenous probiotic strains derived from a specific population might be more suitable for a specific ethnic group. Accordingly, *Lacticaseibacillus casei* (formerly *Lactobacillus casei*) strain T21 isolated from a human gastric biopsy might be suitable for use as a probiotic for populations in Southeast Asia. An investigation of the effect of *L. casei* T21 on *C. difficile* infection in a mouse model and in colonic epithelial cells was conducted.

## Materials and Methods

### Bacterial Strains and Culture Conditions

*L. casei* strain T21 was obtained from the stock culture of the Department of Microbiology, Faculty of Medicine, Chulalongkorn University. Bacterial stock culture was maintained in deMan Rogosa Sharpe (MRS) broth (Oxoid, Hampshire, United Kingdom) containing 20% (vol/vol) glycerol at –80°C. *L. casei* T21 was cultured on MRS agar under anaerobic conditions using gas generation sachets (Anaero Pack-Anaero, Mitsubishi Gas Chemical, Japan) at 37°C for 48 h. *C. difficile* ATCC BAA1870 (ATCC, Manassas, VA, United States) was cultured anaerobically on Brucella agar (Becton Dickinson, France) supplemented with 5% (vol/vol) sheep blood at 37°C for 48 h.

### *C. difficile* Infection Mouse Model and *L. casei* T21 Intervention

**FIGURE 1:**
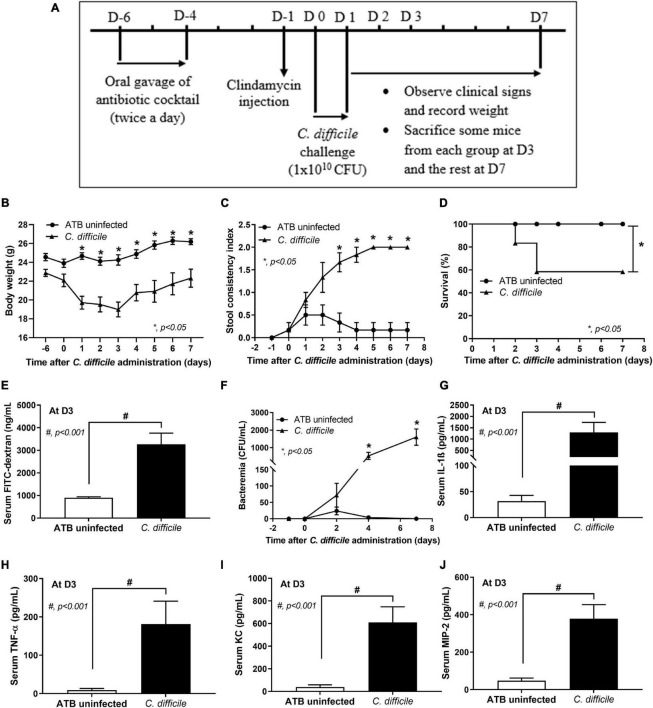
Murine model of *C. difficile* infection. Mice in the antibiotic-administered uninfected group (ATB uninfected group; *n* = 12) and *C. difficile*-infected group (*C. difficile* group; *n* = 24) were used in the experiment as in the schematic presentation **(A)**. The severity of *C. difficile* infection in mice is manifested by weight loss (n = number of mice/group or survivors) **(B)**; stool consistency index (n = number of mice/group or survivors) **(C)**; survival rate **(D)**; bacteremia (n = number of mice/group or survivors) **(E)**; gut leakage by serum FITC-dextran assay monitored at day 3 (*n* = 8) **(F)**; and levels of serum pro-inflammatory cytokines IL-1β **(G)**, TNF-α **(H)**, KC **(I)**, and MIP-2 **(J)** at day 3 (*n* = 8). *p < 0.05; ^#^*p* < 0.001. FITC, fluorescein isothiocyanate; IL, interleukin; TNF, tumor necrosis factor; KC, keratinocyte chemoattractant; MIP-2, macrophage inflammatory protein 2.

The experimental protocol in accordance with the US National Institutes of Health standards (NIH publication no. 85–23, revised 1985) was approved by the Institutional Animal Care and Use Committee of the Faculty of Medicine, Chulalongkorn University (SST006/2560). Male 8-week-old C57BL/6 mice were purchased from the Nomura Siam International Co., Ltd. (Lumphini, Pathumwan, Bangkok, Thailand). CDI mouse model as previously developed (Chen et al., 2008) and recently published (Panpetch et al., 2019) was performed with modifications. Briefly, 500 μL of the antibiotic cocktail (Sigma-Aldrich, St. Louis, MO, United States) containing gentamicin (3.5 mg/kg), colistin (4.2 mg/kg), metronidazole (21.5 mg/kg), and vancomycin (4.5 mg/kg) was administered by oral gavage twice a day from day –6 to day –4 before *C. difficile* infection (D-6–D-4) (Figure 1A). Mice were free from antibiotic administration for 2 days and received an intraperitoneal injection of a single dose of clindamycin (10 mg/kg) at 1 day before infection (D-1). After the treatment with antibiotics (ATB), mice were gavaged with either 0.5 ml of normal saline solution (NSS) in the ATB-administered uninfected group (ATB uninfected group; *n* = 12) or 1 × 10^10^ colony forming units (CFU) of *C. difficile* vegetative cells in 0.5 ml of NSS once daily for 2 days (D0 and D1) in the *C. difficile* group (*n* = 24). Mice were observed and monitored daily for weight, stool consistency, and survival until D7. Blood was collected through tail vein nicking for enumeration of bacteria at D2, D4, and D7. According to our pilot study, *C. difficile*-infected mice developed severe symptoms for 3 days, some succumbed to infection, and the survivors gradually recovered from CDI. At D3, mice from each group (*n* = 8) were then tested for gut leakage and sacrificed with cardiac puncture under isoflurane anesthesia for determining CDI severity by using serum pro-inflammatory cytokines as markers. The stool consistency was semi-quantitatively evaluated using the following scoring; 0, normal; 1, soft or loose; and 2, diarrhea, as previously published (Kim et al., 2012).

**FIGURE 2:**
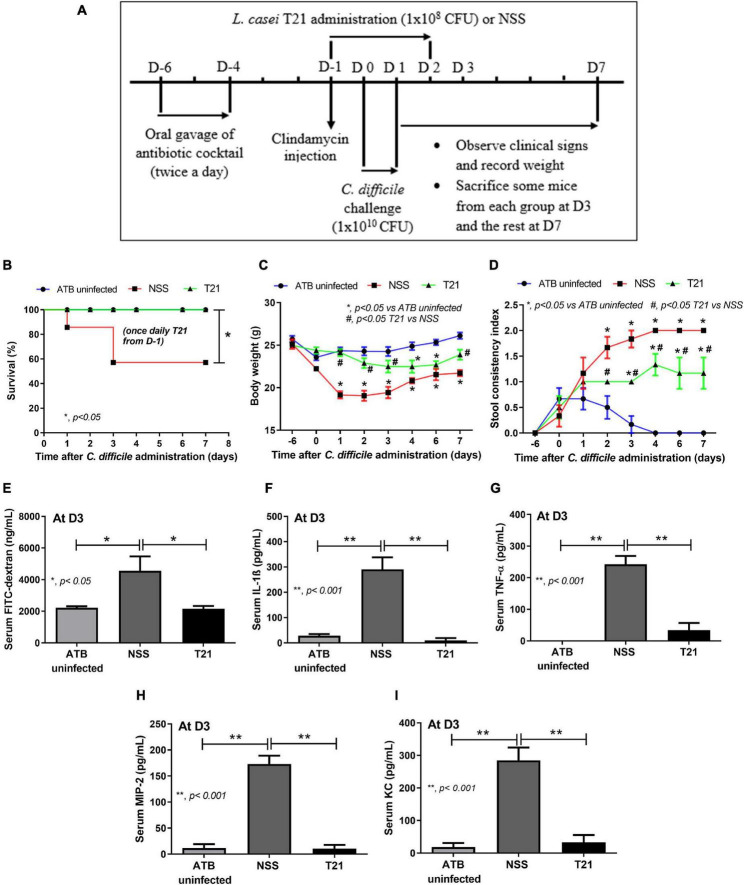
*Lacticaseibacillus casei* T21 effectively attenuated *C. difficile*-induced severity in mice. Mice were randomly assigned to three groups: ATB uninfected group (*n* = 18), NSS group (*n* = 24), and T21 group (*n* = 20) as described in the text and used in the experiment **(A)**. The reduction of severity was demonstrated by survival rate **(B)**; body weight (n = number of mice/group or survivors) **(C)**; stool consistency index (n = number of mice/group or survivors) **(D)**; gut leakage by serum FITC-dextran assay monitored at day 3 (*n* = 8) **(E)**; and levels of serum pro-inflammatory cytokines IL-1β **(F)**, TNF-α **(G)**, MIP-2 **(H)**, and KC **(I)** at day 3 (*n* = 8). *p < 0.05; **p < 0.001; ^#^*p* < 0.05.

For *Lacticaseibacillus* treatment in the CDI mouse model, mice were randomly divided into three groups: ATB uninfected group (*n* = 18), *C. difficile*-infected mice treated with NSS (NSS group; *n* = 24), and *C. difficile*-infected mice treated with *L. casei* T21 (T21 group; *n* = 20), which received 1 × 10^8^ CFU of *L. casei* T21 in 0.5 ml of NSS once daily for 4 days from D-1 (started at 6 h after clindamycin injection), D0 and D1 (together with *C. difficile*), and D2 (Figure 2A). Mice were observed and monitored daily until D7 as described above. Feces were collected for microbiome analysis at D-6 for baseline and D-1 (before clindamycin injection and gavage with *L. casei* T21) as time of post-ATB administration. At D3, some mice from each group (*n* = 8) were tested for gut leakage and sacrificed for determining the parameters of CDI severity. Blood samples and cecal and ascending colonic tissues were collected for the assessment of cytokine levels, which represented systemic and local inflammation, respectively. Cecal and ascending colonic tissues were also used for histopathologic evaluation, while luminal content including feces in the cecum and colon were used for quantitation of *C. difficile* and microbiome analysis. All mice were sacrificed on D7 at the end of experiment.

### Mouse Sample Analysis

The pro-inflammatory cytokines as previously described (Leffler and Lamont, 2015), including IL-1β, tumor necrosis factor (TNF)-α, macrophage inflammatory protein 2 (MIP-2), and keratinocyte chemoattractant (KC), in serum and homogenized tissue of the cecum and colon were measured by an enzyme-linked immunosorbent assay (ELISA) (PeproTech, NJ, United States). For determining tissue pro-inflammatory cytokines, tissue samples (approximately 100 mg) were weighed and homogenized using an Ultra-Turrax homogenizer (IKA, Staufen, Germany) in 500 μL of phosphate-buffered saline (PBS, pH 7.4) containing protease inhibitor and centrifuged at 12,000 × *g* for 15 min at 4°C to separate the supernatant for analysis.

For quantitation of *C. difficile* in luminal content including feces, quantitative real-time polymerase chain reaction (qPCR) was performed as previously published (Panpetch et al., 2019). Briefly, genomic DNA of *C. difficile* was extracted from cecum and colon contents using the High Pure PCR Template Preparation Kit (Roche, NJ, United States), quantified by NanoDrop™ 1000 Spectrophotometer (Thermo Fisher Scientific, Inc., United States), and amplified with *tcdB* (*C. difficile* toxin B) primers (forward, 5′-GGAAAAGAGAATGGTTTTATTAA-3′ and reverse, 5′-ATC TTTAGTTATAACTTTGACATCTTT-3′) as previously described (Lemee et al., 2004) in the QuantStudio 5 Real-Time PCR System (Thermo Fisher Scientific, Inc., United States) using the QuantiNova^®^ SYBR^®^ Green PCR Kit (QIAGEN, Hilden, Germany). The standard curve was created by using 10-fold serially diluted plasmids containing 1–10^9^ copies of *tcdB*. The number of *C. difficile* was calculated using the standard curve and shown as bacterial copy number.

Histopathological analysis was performed by fixing the sample in 10% buffered formalin, embedding in paraffin, sectioning, and staining with hematoxylin and eosin (H&E) before grading (score 0–4) as previously published (Reeves et al., 2011; Erikstrup et al., 2015) based upon epithelial damage, edema, and cellular infiltration by two pathologists in a blinded manner.

### Microbiome Analysis

Gut microbiota composition was determined as previously reported (Panpetch et al., 2020). Feces collected at D-6 as baseline, D-1 as time of post-ATB administration, and luminal content including feces at D3 as time of sacrifice were used for microbiome analysis. Briefly, fecal samples or luminal content (0.25 g/mouse; three mice/group) were extracted for total DNA with the DNeasy PowerSoil Kit (Qiagen GmbH, Hilden, Germany). The quality and concentration of the extracted DNA were monitored by agarose gel electrophoresis and NanoDrop spectrophotometry. Libraries of the V4 hypervariable region of 16S rRNA gene were amplified by PCR using primers 515F (forward; 5′-GTGCCAGCMGCCGCGGTAA-3′) and 806R (reverse; 5′-GGACTACHVGGGTWTCTAAT-3′), modified with the Illumina adapter and Golay barcode sequences as previously described (Caporaso et al., 2012). PCR was run in triplicate, and the products from the triplicate reactions were pooled and visualized on agarose gel. Amplicons of approximately 381 base pairs were purified by PureDireX PCR Clean-Up & Gel Extraction Kit (BIO-HELIX Co., Ltd., Keelung City, Taiwan) and quantified using PicoGreen fluorescence with the Qubit dsDNA HS assay kit (Invitrogen, Eugene, OR, United States). The amplicon pool was sequenced with the Illumina MiSeq300 platform (Illumina, San Diego, CA, United States) (Caporaso et al., 2012). Sequences were analyzed with Mothur version 1.3 (Schloss et al., 2009). Briefly, quality filtering and trimming were performed to remove low-quality bases and short reads from the raw sequences. Quality-filtered sequences were then aligned to each other and binned into operational taxonomic units (OTUs) with a minimum of 97% similarity. Each representative OTU sequence was compared to the SILVA rDNA sequence database (version 1.32) and assigned a taxonomical annotation. Alpha diversity (total OTUs, Chao1 index, and Shannon diversity) and beta diversity (non-metric multidimensional scaling) were calculated using Mothur (Schloss et al., 2009). The 16S rDNA sequences in this study were deposited in an NCBI open access Sequence Read Archive database with accession number SRP336496.

### Gut Leakage Measurement and Enumeration of Bacteria in the Blood

Intestinal epithelial permeability defect (gut leakage) was determined using a single oral administration of 12.5 mg fluorescein isothiocyanate–dextran (FITC-dextran; molecular weight 4.4 kDa) (Sigma-Aldrich, St. Louis, MO, United States), a non-intestinal-absorbable marker, before the determination in serum at 3 h later as previously described (Leelahavanichkul et al., 2016; Panpetch et al., 2018). Serum FITC-dextran was measured by the fluorospectrometry (Thermo Fisher Scientific, Wilmington, DE, United States) with the excitation and emission wavelengths at 485 and 523 nm, respectively, against a standard curve of serially diluted FITC-dextran. For the enumeration of live bacteria, blood (25 μL) was collected through tail vein nicking and spread directly onto blood agar (Oxoid, Hampshire, United Kingdom) and incubated at 37°C for 24 h before counting bacterial colonies.

### The Immunomodulatory Effect of *L. casei* T21 on *C. difficile-*Stimulated Colonic Epithelial Cells

The conditioned medium of *L. casei* T21 was tested for immunomodulation of IL-8 production in colonic epithelial cell lines as previously described (Panpetch et al., 2016, 2018). In brief, *Lacticaseibacillus*-conditioned medium (LCM) was prepared by growing *L. casei* T21 with an OD_600_ of 0.1 in MRS broth anaerobically for 48 h. The supernatant was collected and subjected to filtration with a 0.22-μm membrane (Minisart, Sartorius Stedim Biotech GmbH, Goettingen, Germany), concentrated by speed vacuum drying, resuspended in cell culture medium with equal volume, and stored at –20°C until use. In parallel, human colonic epithelial cell lines Caco-2 (ATCC HTB-37) and HT-29 (ATCC HTB-38) were maintained (5 × 10^4^ cells/well) in supplemented Dulbecco’s Modified Eagle Medium (DMEM) and McCoy’s 5A modified medium, respectively. Colonic epithelial cells were then incubated with viable cells of *C. difficile* ATCC BAA1870 at multiplicity of infection (MOI) 1:300 either alone or with 5% (vol/vol) LCM for 24 h in 5% CO_2_ at 37°C. Subsequently, the supernatant was collected by centrifugation (125 × *g*, 4°C for 7 min), and the levels of IL-8 were measured by using a Human CXCL8/IL-8 ELISA kit (R&D Systems, Minneapolis, MN) according to the manufacturer’s instructions.

In addition, colonic epithelial cells at 2 and 4 h from the incubation time were collected for performing quantitative reverse transcription-polymerase chain reaction (qRT-PCR) as previously described (Panpetch et al., 2018). In short, the total RNA of treated colonic epithelial cells was extracted by TRIzol reagent (Invitrogen, United States), prepared for complementary DNA (cDNA) from total RNA (50 ng) by SuperScript^®^ VILO™ cDNA Synthesis Kit (Invitrogen), and subjected to qPCR measurement in a QuantStudio™ Design & Analysis Software v1.4.3 (Thermo Fisher Scientific) with the following primers: IL-8 (forward 5′-ACACTGCGCCAACACAGAAATTA-3′, reverse 5′-ACACTGCGCCAACACAGAAATTA-3′) and human glyceraldehyde-3-phosphate dehydrogenase (GAPDH) (forward 5′-GCACCGTCAAGGCTGAGAAC-3′, reverse 5′-ATGGTGGTGAAGACGCCAGT-3′) (Imaoka et al., 2008; Panpetch et al., 2016). The expression of *IL-8* relative to *GAPDH* was calculated according to the 2^−ΔΔ*Cp*^ method (Pfaffl, 2001).

### The Effect of *L. casei* T21 on the Expression of *SLC11A1, HuR, and MUC2 in C. difficile-*Stimulated Colonic Epithelial Cells

The conditioned medium of *L. casei* T21 was tested for its effect on the expression of *C. difficile-*activated host genes *SLC11A1*, *HuR*, and *MUC2* by using qRT-PCR as described above with the following primers: SLC11A1 (forward 5′-CTGGACGAATCCCACTCTGG-3′, reverse 5′-CGCGCCACCACATACTCAT-3′), HuR (forward 5′-GCTTGGGCTATGGCTTTGTGAACT-3′, reverse 5′-CGCTG ATGTACAAGTTGGCGTCTT-3′) (Feng and Cohen, 2013), and mucin2 (MUC2) (forward 5′-CCTGCCGACACCTGCTGCAA-3′, reverse 5′-ACACCAGTAGAAGGGACAGCACCT-3′) (Xue et al., 2014). In parallel, the pH of cell culture medium was measured at multiple time points using a pH meter (Orion 4-star, Benchtop pH/Conductivity, Thermo Fisher Scientific).

### The Effect of *L. casei* T21 on Transepithelial Electrical Resistance of Caco-2 Cells

TEER was performed according to a previous report (Gao et al., 2017). In short, Caco-2 cells (ATCC HTB-37) at 5 × 10^4^ cells per well were seeded onto the upper compartment of a 24-well Boyden chamber transwell using high-glucose DMEM supplemented with 20% fetal bovine serum (FBS), 1% HEPES, 1% sodium pyruvate, and 1.3% penicillin/streptomycin under 5% CO_2_ at 37°C for 15 days with daily medium replacement to establish the confluent monolayer. The cells were then treated with 5% (vol/vol) LCM of *L. casei* T21 or medium alone together with viable *C. difficile* cells (5 × 10^6^ CFU/well) with MOI at 1:100 for 24 h. Next, TEER was measured by an EMOM^2^ Epithelial Volt/Ohm Meter with a chopstick electrode (World Precision Instruments, Inc., Sarasota, United States) that was placed at a 90° angle with one tip in supernatant at the basolateral chamber and another tip at the apical chamber. The TEER value in control media without cells was used as a baseline subtracted from all measurements. The value of TEER was reported as ohm (Ω) × cm^2^.

### Statistical Analysis

Mean ± standard error of mean (SEM) was used for data presentation. The difference between groups was examined for statistical significance by one-way analysis of variance (ANOVA) followed by Tukey’s analysis or unpaired *t* tests for comparisons of multiple groups or two groups, respectively. Survival analysis was performed by log-rank test. All statistical analyses were performed with GraphPad Prism version 9.0 software (La Jolla, CA, United States). A *p*-value of < 0.05 was considered statistically significant.

## Results

### Disease Progression and Severity of Murine *C. difficile* Infection Model

In the CDI murine model (Figure 1A), *C. difficile*-infected mice began to develop CDI symptoms such as weight loss and soft stool on day 1 after the first oral gavage with *C. difficile* on day 0. After the second oral gavage on day 1, CDI symptoms became worse on day 2, and mice were moribund on day 3 with maximum weight loss (Figure 1B) and significant diarrhea (loose stools) as compared with the ATB uninfected group (Figure 1C). By day 3, 41.67% (10/24) of mice succumbed to infection (58.33% survival rate) (Figure 1D). Eight mice were sacrificed on day 3, and the remaining six mice gradually gained weight while still having diarrhea and surviving until the end of the experiment (Figures 1B,C). Additionally, *C. difficile* damaged intestinal integrity as demonstrated by the increased serum FITC-dextran levels (Figure 1E), causing gut leakage-induced bacteremia (Figure 1F) that enhanced the production of systemic inflammatory cytokines (serum IL-1β, TNF-α, KC, and MIP-2 levels as markers) (Figures 1G–J). In contrast, mice in the ATB uninfected group demonstrated loose stools for a few days (days 1–3) without weight loss, death, gut leakage, bacteremia, or systemic inflammation (Figures 1B–J).

### *L. casei* Strain T21 Reduced Mortality, Clinical Symptoms, and Disease Severity of *C. difficile-*Infected Mice

**FIGURE 3:**
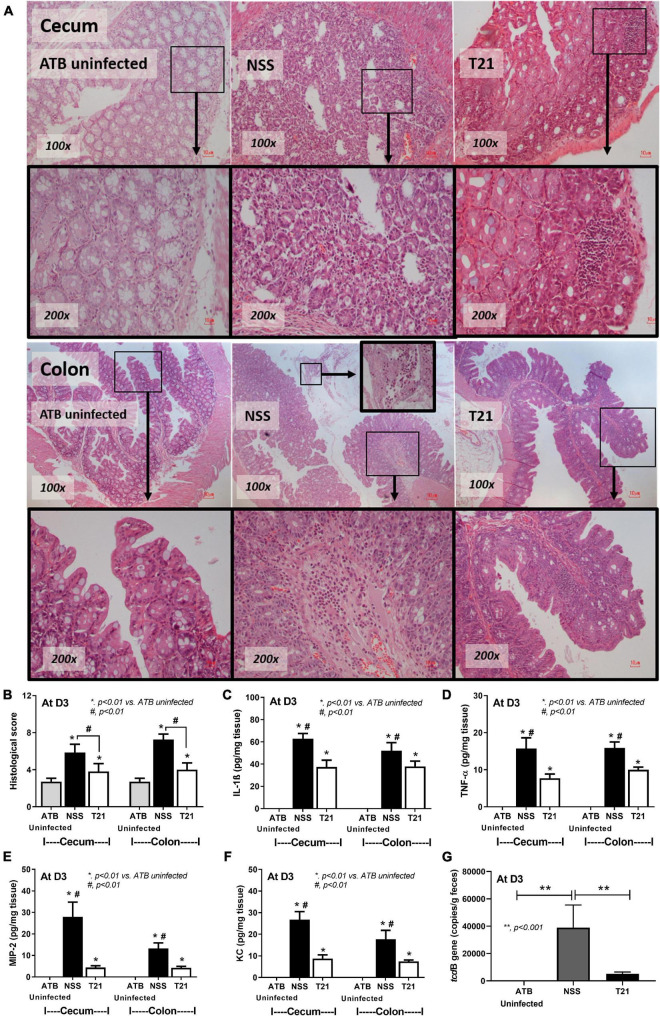
*Lacticaseibacillus casei* T21 attenuated *C. difficile*-induced cecal and colonic tissue damage. Three groups of mice (*n* = 8 for each group) as described in Figure 2 were sacrificed at day 3 and examined for histopathology and local inflammation. Representative image of hematoxylin and eosin (H&E) staining sections **(A)**, histological scores **(B)**, and levels of intestinal pro-inflammatory cytokines **(C–F)** are shown. The copy number of *tcdB*, which represents *C. difficile* abundance in each group of mice, is demonstrated **(G)**. *p < 0.01; **p < 0.001*; ^#^p* < 0.01.

For the treatment of *L. casei* T21 in the murine model of CDI, *C. difficile*-infected mice in the T21 group received 1 × 10^8^ CFU of *L. casei* T21 once daily for 4 days (D-1–D-2), whereas infected mice in the NSS group received NSS (Figure 2A). All mice in the T21 and the ATB uninfected group survived, while only 54.17% (13/24) of the NSS group survived by day 3 (Figure 2B). Compared to the NSS group, the T21 group had significantly lesser weight loss (except at days 4 and 5) (Figure 2C) and diarrhea (Figure 2D), which were monitored for 7 days. The average weight at each day (except at days 4 and 5) between the T21 group and the ATB uninfected group showed no significant difference (Figure 2C). However, mice in the T21 group still had soft stool with a stool consistency index significantly different from the ATB uninfected group (Figure 2D). Treatment with T21 also reduced gut leakage and systemic inflammation. The T21 group had significantly decreased levels of FITC-dextran (Figure 2E) and pro-inflammatory cytokines IL-1β, TNF-α, MIP-2, and KC in sera (Figures 2F–I). Likewise, *L. casei* T21 also attenuated intestinal injury in the cecum and colon as evaluated by histopathology (Figures 3A,B) and reduced the levels of pro-inflammatory cytokines in the intestinal tissue (Figures 3C–F). Notably, *C. difficile*-infected mice without T21 treatment (NSS group) demonstrated several characteristics of severe intestinal injury, including loss of villi, villous edema, numerous neutrophil infiltration (Figure 3A), and neutrophils in feces (data not shown). In addition, T21 treatment reduced the abundance of *C. difficile* in the intestinal content. The analysis of luminal content including feces by q-PCR of the *tcdB* gene demonstrated that the T21 group had significantly decreased copies compared to the NSS group (Figure 3G). Moreover, the absence of the *tcdB* gene in the ATB uninfected mice (Figure 3G) revealed the reliability of the experiment.

### *L. casei* T21 Slightly Attenuated Gut Dysbiosis in *C. difficile-*Infected Mice

**FIGURE 4:**
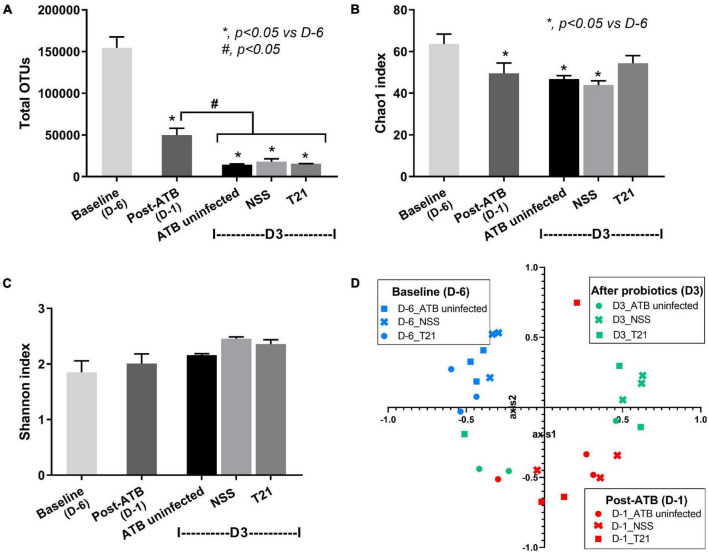
Diversity of gut microbiota in each group of mice at multiple time points. Fecal microbiome data of the ATB uninfected, NSS, and T21 groups (*n* = 3/group) at baseline, post-ATB, and at sacrifice were calculated for microbial diversity and presented as total operational taxonomic units (OTUs) **(A)**, Chao1 index **(B)**, Shannon index **(C)**, and the non-metric multidimensional scaling (NMDS) based on Thetayc dissimilarity **(D)**. Independent triplicate experiments were performed. *p < 0.05 vs. D-6, ^#^*p* < 0.05.

Gut dysbiosis of the model was evaluated by a fecal microbiome analysis of mice in the ATB uninfected, NSS, and T21 groups at multiple time points. Alpha diversity measures used in this study included total OTUs (the simplest measure of richness), Chao1 (a measure of richness that gives more weight to rare taxon), and Shannon (a measure of richness and evenness). Antibiotic cocktail treatment significantly reduced the diversity of fecal bacteria as the values of the total OTUs (Figure 4A) and Chao1 index (Figure 4B) were significantly lower in all groups of mice at post-ATB compared to baseline. After clindamycin injection, antibiotic-induced dysbiosis worsened at day 3 of the experiment as determined by total OTUs (Figure 4A), but not by Chao1 index (Figure 4B). *C. difficile* infection did not lead to a significant decrease of bacterial diversity as demonstrated by total OTUs (Figure 4A) and Chao1 index (Figure 4B). Surprisingly, T21 treatment did not increase the diversity of fecal bacteria as the values of total OTUs (Figure 4A) and Chao1 index (Figure 4B) of the NSS and T21 groups were not significantly different. The T21 group did show an increased Chao1 index, although the value was not significantly different from that of the NSS group. The values of Chao1 index were also not significantly different in the T21 and ATB uninfected groups (Figure 4B). Notably, the bacterial diversity in all groups of mice at day 3, post-ATB, and baseline was not significantly different as determined by the Shannon index (Figure 4C). To examine the beta diversity, non-metric multidimensional scaling (NMDS) based on Thetayc dissimilarity was performed. The NMDS (Figure 4D) demonstrated similar results in all groups at baseline (D-6) (blue-colored symbols at the upper left quadrant) and at post-ATB (D-1) (red-colored symbols at the lower right quadrant). In contrast, there were some differences at D3 from the experiments between groups of mice with or without *L. casei* T21 (green-colored symbols of the NSS and T21 groups), suggesting a possible impact of *L. casei* T21 on gut microbiota. The ATB uninfected and T21 groups showed similar results.

**FIGURE 5:**
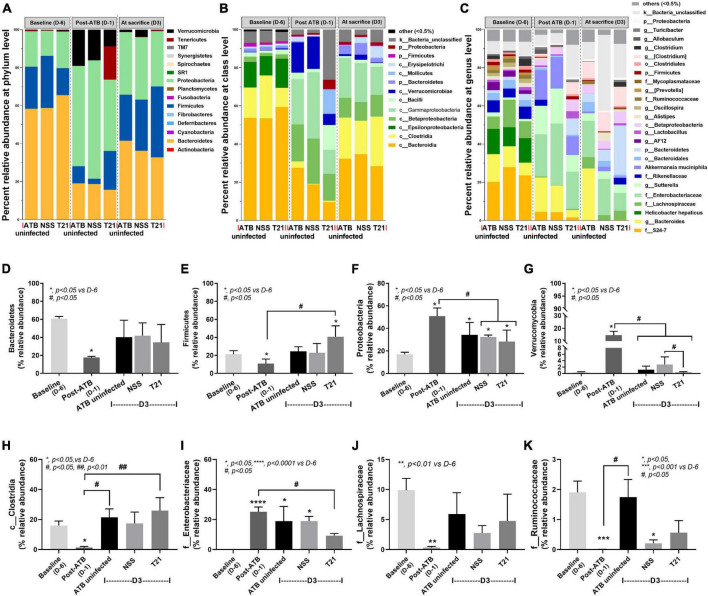
*Lacticaseibacillus casei* T21 slightly attenuated gut dysbiosis in *C. difficile*-infected mice. Gut dysbiosis of the *C. difficile* infection (CDI) model was evaluated by fecal microbiome analysis of mice in the ATB uninfected, NSS, and T21 groups at multiple time points. The average relative abundances of microbiota at the phylum level **(A)**, class level **(B)**, and genus level **(C)** are demonstrated. The average relative abundances of individual taxon are shown: Bacteroidetes **(D)**, Firmicutes **(E)**, Proteobacteria **(F)**, Verrucomicrobia **(G)**, Clostridia **(H)**, *Enterobacteriaceae* **(I)**, *Lachnospiraceae* **(J)**, and *Ruminococaceae* **(K)**. Notably, data of all mice at day –6 (D-6) and day –1 (D-1) of the experiments are combined into baseline and post-antibiotic administration (post-ATB), respectively, due to the non-different procedures in these mice. *p < 0.05; **p < 0.01; ***p < 0.001; ****p < 0.0001; ^#^*p* < 0.01; ^##^*p* < 0.01.

To characterize the microbiome composition in each group of mice, the relative taxa abundances at each time point were compared. The gut microbiome of mice at baseline was predominated by Bacteriodetes, followed by Firmicutes and Proteobacteria, as shown by the average relative abundances of microbiota at the phylum level in each group of mice (Figure 5A) and the relative abundances of Bacteroidetes (Figure 5D), Firmicutes (Figure 5E), and Proteobacteria (Figure 5F) at each time point. In contrast, antibiotic cocktail treatment induced a significant decrease in the relative abundance of Bacteriodetes and Firmicutes and a significant increase in the abundance of Proteobacteria and Verrucomicrobia at D-1 (Figures 5A,D–G). Antibiotic pre-conditioning of the model thus caused fecal dysbiosis as indicated by a decrease in Firmicutes and Bacteroidetes together with an increase in Proteobacteria and Verrucomicrobia. By day 3, microbiota composition at the phylum level almost turned to the baseline without T21 treatment. The relative abundance of Bacteroidetes (Figure 5D), Firmicutes (Figure 5E), and Verrucomicrobia (Figure 5G) in the ATB uninfected group and the NSS group was not significantly different from the baseline, whereas the abundance of Proteobacteria was still significantly different from the baseline (Figure 5F). *L. casei* T21 treatment led to an increase in the relative abundance of Firmicutes in the T21 group with a significant difference compared to post-ATB, but not from the NSS group (Figure 5E). In addition, T21 treatment resulted in a significant decrease in the relative abundance of Verrucomicrobia in the T21 group compared to the NSS group and post-ATB, and the abundance of Verrucomicrobia in the T21 group was not significantly different from the baseline (Figure 5G). Although the relative abundance of Proteobacteria in the T21 group significantly decreased compared to post-ATB, this change also occurred in the NSS group (Figure 5F).

The analysis of microbiome at the lower taxon levels (class, order, family, genus, and species) was performed. The average relative abundances of microbiota at baseline, post-ATB, and among groups of mice at sacrifice (day 3) are shown at the class level (Figure 5B) and the genus level (Figure 5C). There were significant changes in class Clostridia (Figure 5H) and family *Enterobacteriaceae* (Figure 5I) in the T21 group at sacrifice (D3) as compared with mice at post-ATB. Although the NSS group had changes in the relative abundance of these taxa as compared with mice at post-ATB, the differences were not statistically significant. Microorganisms of interest in class Clostridia (phylum Firmicutes) were the families *Lachnospiraceae* and *Ruminococcaceae* (Figure 5C) that have been reported to protect *C. difficile* colonization (Reeves et al., 2012; Lee et al., 2017). The relative abundances of the family *Lachnospiraceae* (Figure 5J) were not significantly different among the ATB uninfected, the NSS, and the T21 groups, while there was a slight increase in the abundance of the family *Ruminococcaceae* in the T21 group compared to the NSS group, although the difference was not statistically significant (Figure 5K). The relative abundances of other taxa (at the class and genus levels) were also not significantly different among these groups of mice at sacrifice (data not shown). Data on the relative abundances of microbiota from individual mouse are shown at the levels of phylum (Supplementary Figure 1), class (Supplementary Figure 2), and genus (Supplementary Figure 3) in Supplementary Material.

### *L. casei* T21 Suppressed IL-8 Production, Modulated Host Gene Expression, and Increased Transepithelial Electrical Resistance of *C. difficile-*Activated Colonic Epithelial Cells

**FIGURE 6:**
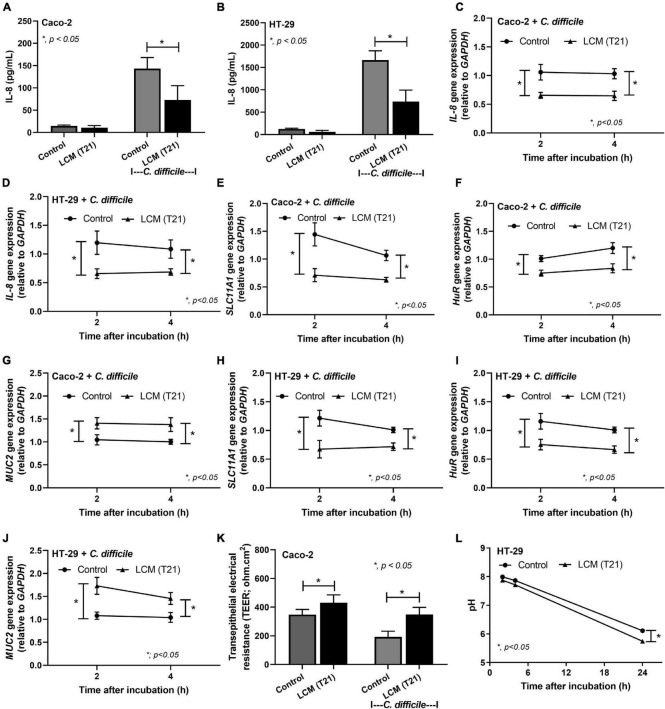
The *Lacticaseibacillus casei*-conditioned medium (LCM) of *L. casei* T21 attenuated *C. difficile*-induced IL-8 production, host gene expression, and increased transepithelial electrical resistance of *C. difficile*-stimulated colonic epithelial cells. The results are shown as IL-8 production in Caco-2 and HT-29 cells, respectively **(A,B)**; IL-8 gene expression (relative to *GAPDH*) in Caco-2 and HT-29 cells, respectively **(C,D)**; the expression of *SLC11A1*, *HuR*, and *MUC-2* in Caco-2 cells, respectively **(E–G)**; the expression of *SLC11A1*, *HuR*, and *MUC-2* in HT-29 cells, respectively **(H–J)**; the transepithelial electrical resistance (TEER) values of Caco-2 cells **(K)**; and the pH of cell culture medium (McCoy’s 5A modified medium for HT-29 cells) **(L)**. The results were from three independent experiments each in triplicate and expressed as the mean ± SEM. *p < 0.05.

Probiotic bacteria, such as *Lactobacillus* spp., have been reported to produce biologically active compounds that can suppress inflammation (Thomas et al., 2012, 2016; Panpetch et al., 2016, 2018). To strengthen the beneficial effects of *L. casei* T21 in the murine model of CDI, the LCM of T21 was tested for its ability to attenuate inflammation and modulate the expression of important host genes involved in the pathogenesis of CDI in colonic epithelial cell lines. The LCM of T21 suppressed the production of IL-8 (Figures 6A,B) and down-regulated the expression of *IL-8* (Figures 6C,D) in *C. difficile*-stimulated Caco-2 and HT-29 colonic epithelial cells, respectively. In *C. difficile*-stimulated Caco-2 cells, the LCM of T21 down-regulated the expression of associated genes of toxin lethality *SLC11A1* (Figure 6E) and *HuR* (Figure 6F) while up-regulated a mucosal protective gene *MUC2* (Figure 6G). Similar benefits exerted by the LCM of T21 were also demonstrated in HT-29 cells (Figures 6H–J). Additionally, the LCM of T21 strengthened mucosal integrity as shown by TEER values in *C. difficile*-stimulated differentiated Caco-2 cells (Figure 6K). However, TEER could not be determined in the HT-29 cell line due to limitation in the generation of polarized monolayers (Le Bivic et al., 1988). Since the enhanced mucin production by enterocytes is also influenced by the acidity in gut content (Velcich and Augenlicht, 1993; Shekels et al., 1996), lactic acid produced from *L. casei* T21 might stimulate the up-regulation of *MUC2* expression. To determine whether the up-regulated *MUC2* could be attributable to the acidity, the pH of the cell culture medium (McCoy’s 5A modified medium) was measured at multiple time points. The LCM of T21 did not significantly reduce the pH of the cell culture medium at 2 and 4 h after incubation with HT-29 cells as compared to controls (Figure 6L). A simple acidification of the cell culture medium with lactic acid also did not upregulate *MUC2* expression (data not shown). This suggested that mucin production was induced by other substances produced by *L. casei* T21.

## Discussion

In this study, the effect of *L. casei* T21 treatment on CDI was investigated in a murine model of *C. difficile* infection with antibiotic pre-conditioning before *C. difficile* oral gavage. The clinical features of CDI are primarily mediated by TcdA and TcdB, which are the major virulence factors of *C. difficile* (Voth and Ballard, 2005; Sun et al., 2010). Clinical symptoms including less weight loss and diarrhea in *L. casei* T21-treated mice are correlated with mild intestinal pathology characterized by decreased epithelial damage, edema, and neutrophil infiltration. Due to the importance of intestinal neutrophil accumulation in *C. difficile* pathogenesis, the anti-inflammatory properties of probiotics is of utmost importance for the alleviation of CDI symptoms. Neutrophil infiltration, which leads to the congestion and edema of colonic mucosa and epithelial cell damage (Kelly et al., 1994a; Castagliuolo et al., 1998), results from the stimulation by pro-inflammatory cytokines secreted from *C. difficile* toxin-induced intestinal epithelial cells and immune cells (Kim et al., 2002; Savidge et al., 2003; Sun et al., 2010). Interestingly, *L. casei* T21 administration markedly reduced the levels of IL-1β, TNF-α, MIP-2, and KC (mouse homolog of IL-8) in the colon and cecum of mice as well as in sera. The anti-inflammatory properties of *L. casei* T21was also supported by *in vitro* results that found the conditioned medium of *L. casei* T21 suppressed gene expression and production of IL-8 in *C. difficile*-stimulated colonic epithelial cells Caco-2 and HT-29. Our findings were consistent with other reports showing that the administration of specific strains of probiotics ameliorates intestinal pathology with the reduction in the levels of pro-inflammatory cytokines in tissue and/or sera in animal models of CDI (Koon et al., 2016; Roychowdhury et al., 2018; Wei et al., 2018; Xu et al., 2018). The production of anti-inflammatory substances from lactobacilli is well known (Thomas et al., 2012, 2016; Boonma et al., 2014; Panpetch et al., 2016, 2018), although the nature of the substances varies depending on the strain of probiotic bacteria.

Our microbiome results regarding antibiotic-induced dysbiosis is in agreement with findings from previous reports (Mooyottu et al., 2017; Xu et al., 2018; Li et al., 2019) that showed antibiotic treatment induced a decrease in the dominant bacterial phyla Firmicutes and Bacteroidetes and an increase in phylum Proteobacteria. Although the influence of *L. casei* T21 on gut dysbiosis attenuation was subtle, with an increase in members of phylum Firmicutes (class Clostridia) and a decrease in family *Enterobacteriaceae* (phylum Proteobacteria), the abundance of *C. difficile* in cecum and colon luminal content was decreased as determined by quantitative analysis of *C. difficile* toxin B gene (*tcdB*). Members of class Clostridia, which have been reported to protect *C. difficile* colonization, include the family *Lachnospiraceae* (Reeves et al., 2012; Lee et al., 2017) and the family *Ruminococcaceae* (Lee et al., 2017; Li et al., 2019). However, our results showed only a slight increase in *Ruminococcaceae* in mice treated with *L. casei* T21. We speculate that members of class Clostridia either alone or in combination might mediate colonization resistance against *C. difficile*.

Since *C. difficile* toxins are mainly responsible for the pathogenesis of CDI, an interference in toxin effect might be another mechanism to be considered for *C. difficile* attenuation (Kolling et al., 2012; Panpetch et al., 2019; Yong et al., 2019). *C. difficile* toxins inactivate Rho GTPases resulting in gut leakage, intestinal inflammation, and cell death (Chen et al., 2015). The interference with the expression of toxin lethality-associated genes *SLC11A1* and *HuR* may lead to the reduction of gut leakage, inflammation, and mortality in *C. difficile*-infected mice treated with *L. casei* T21. While *L. casei* T21 reduced the toxin effect by down-regulation of the expression of *SLC11A1* and *HuR*, which enhances TcdB action, other probiotics interfere with the activity of *C. difficile* toxins by other mechanisms. For example, *Saccharomyces boulardii* interferes the binding between the toxins and intestinal brush borders (Castagliuolo et al., 1999), *L. delbrueckii* directly inhibits the cytotoxicity (Banerjee et al., 2009), and *Streptococcus thermophilus* reduced toxin production through potent lactic acid generation (Kolling et al., 2012).

*L. casei* T21 was found to up-regulate the expression of *MUC2*, which codes for mucin, an intestinal mucosal protective factor, referred to as “mucin barrier” (Dharmani et al., 2009; Paone and Cani, 2020), which can promote gut integrity. Similarly, *Lactobacillus plantarum* induces the expression of *MUC2* and *MUC3* that inhibit *Escherichia coli* adherence to intestinal epithelium cells (Mack et al., 1999). The mucin-binding protein in several strains of lactobacilli also implies an association between lactobacilli and intestinal mucin (Cornick et al., 2015). Although the enterocyte stimulation by lactic acid might theoretically enhance mucin production (Shekels et al., 1996), the direct incubation of lactic acid-containing cell culture medium with HT-29 cell line did not result in the up-regulation of *MUC2* expression. Other substances produced by *L. casei* T21 are possibly associated with *MUC2* gene up-regulation. More studies on this topic are required. Our findings revealed that *L. casei* T21 had a protective effect against *C. difficile* infection and suggested a great potential of *L. casei* T21 as a probiotic for humans, especially in Southeast Asian populations.

## Conclusion

Our study demonstrated that *L. casei* strain T21 attenuated *C. difficile* infection in mice through anti-inflammation, attenuation of gut leakage and dysbiosis, interference with toxin lethality by down-regulation of the toxin enhancer gene, and augmentation of mucin production by up-regulation of mucin-producing gene.

## Data Availability Statement

The datasets presented in this study can be found in online repositories. The names of the repository/repositories and accession number(s) can be found below: NCBI (accession: SRP336496).

## Ethics Statement

The animal study was reviewed and approved by the experimental protocol in accordance with the US National Institutes of Health standards (NIH publication No. 85-23, revised 1985) was approved by the Institutional Animal Care and Use Committee of the Faculty of Medicine, Chulalongkorn University (SST006/2560).

## Author Contributions

ST, AL, and WP designed the study. WP performed *in vitro* and *in vivo* experiments. PP performed *in vitro* experiments. WP, TC, and NS designed and performed the microbiome analysis. WP, AL, and ST analyzed the data, discussed the results, and wrote the manuscript. All authors contributed to the article and approved the submitted version.

## Conflict of Interest

The authors declare that the research was conducted in the absence of any commercial or financial relationships that could be construed as a potential conflict of interest.

## Publisher’s Note

All claims expressed in this article are solely those of the authors and do not necessarily represent those of their affiliated organizations, or those of the publisher, the editors and the reviewers. Any product that may be evaluated in this article, or claim that may be made by its manufacturer, is not guaranteed or endorsed by the publisher.

## References

[B1] Aktories K. Barbieri J. T. (2005). Bacterial cytotoxins: targeting eukaryotic switches. *Nat. Rev. Microbiol.* 3 397–410. 15821726 10.1038/nrmicro1150

[B2] Aktories K. Papatheodorou P. Schwan C. (2018). Binary *Clostridium difficile* toxin (CDT) - A virulence factor disturbing the cytoskeleton. *Anaerobe* 53 21–29. 10.1016/j.anaerobe.2018.03.001 29524654

[B3] Aslam S. Hamill R. J. Musher D. M. (2005). Treatment of *Clostridium difficile*-associated disease: old therapies and new strategies. *Lancet Infect. Dis.* 5 549–557. 10.1016/S1473-3099(05)70215-2 16122678

[B4] Banerjee P. Merkel G. J. Bhunia A. K. (2009). *Lactobacillus delbrueckii* ssp. bulgaricus B-30892 can inhibit cytotoxic effects and adhesion of pathogenic *Clostridium difficile* to Caco-2 cells. *Gut Pathog.* 1:8. 10.1186/1757-4749-1-8 19397787 PMC2680912

[B5] Bartlett J. G. (2002). Clinical practice. Antibiotic-associated diarrhea. *N. Engl. J. Med.* 346 334–339. 11821511 10.1056/NEJMcp011603

[B6] Boonma P. Spinler J. K. Venable S. F. Versalovic J. Tumwasorn S. (2014). *Lactobacillus rhamnosus* L34 and *Lactobacillus casei* L39 suppress *Clostridium difficile*-induced IL-8 production by colonic epithelial cells. *BMC Microbiol.* 14:177. 10.1186/1471-2180-14-177 24989059 PMC4094603

[B7] Caporaso J. G. Lauber C. L. Walters W. A. Berg-Lyons D. Huntley J. Fierer N. (2012). Ultra-high-throughput microbial community analysis on the Illumina HiSeq and MiSeq platforms. *ISME J.* 6 1621–1624. 22402401 10.1038/ismej.2012.8 PMC3400413

[B8] Castagliuolo I. Keates A. C. Wang C. C. Pasha A. Valenick L. Kelly C. P. (1998). *Clostridium difficile* toxin A stimulates macrophage-inflammatory protein-2 production in rat intestinal epithelial cells. *J. Immunol.* 160 6039–6045. 9637520

[B9] Castagliuolo I. Riegler M. F. Valenick L. Lamont J. T. Pothoulakis C. (1999). *Saccharomyces boulardii* protease inhibits the effects of *Clostridium difficile* toxins A and B in human colonic mucosa. *Infect. Immun.* 67 302–307. 10.1128/IAI.67.1.302-307.1999 9864230 PMC96311

[B10] Chen S. Sun C. Wang H. Wang J. (2015). The role of Rho GTPases in toxicity of *Clostridium difficile* toxins. *Toxins (Basel*) 7 5254–5267. 10.3390/toxins7124874 26633511 PMC4690124

[B11] Chen X. Katchar K. Goldsmith J. D. Nanthakumar N. Cheknis A. Gerding D. N. (2008). A mouse model of *Clostridium difficile*-associated disease. *Gastroenterology* 135 1984–1992. 10.1053/j.gastro.2008.09.002 18848941

[B12] Cornick S. Tawiah A. Chadee K. (2015). Roles and regulation of the mucus barrier in the gut. *Tissue Barriers* 3:e982426. 10.4161/21688370.2014.982426 PMC4372027 25838985

[B13] Dharmani P. Srivastava V. Kissoon-Singh V. Chadee K. (2009). Role of intestinal mucins in innate host defense mechanisms against pathogens. *J. Innate Immun.* 1 123–135. 20375571 10.1159/000163037 PMC7312850

[B14] Erikstrup L. T. Aarup M. Hagemann-Madsen R. Dagnaes-Hansen F. Kristensen B. Olsen K. E. (2015). Treatment of *Clostridium difficile* infection in mice with vancomycin alone is as effective as treatment with vancomycin and metronidazole in combination. *BMJ Open Gastroenterol.* 2:e000038. 10.1136/bmjgast-2015-000038 26568840 PMC4641438

[B15] FAO and WHO (2001). *Report of a Joint FAO/WHO Expert Consultation on Evaluation of Health and Nutritional Properties of Probiotics in Food Including Powder Milk With Live Lactic Acid Bacteria.* Rome: Food and Agriculture Organization of the United Nations, World Health Organization.

[B16] Feng Y. Cohen S. N. (2013). Upregulation of the host SLC11A1 gene by *Clostridium difficile* toxin B facilitates glucosylation of Rho GTPases and enhances toxin lethality. *Infect. Immun.* 81 2724–2732. 10.1128/IAI.01177-12 23690404 PMC3719560

[B17] Gao Y. Li S. Wang J. Luo C. Zhao S. Zheng N. (2017). Modulation of intestinal epithelial permeability in differentiated Caco-2 cells exposed to Aflatoxin M1 and Ochratoxin A individually or collectively. *Toxins (Basel)* 10:13. 10.3390/toxins10010013 PMC5793100 29280945

[B18] Gerding D. N. Johnson S. Rupnik M. Aktories K. (2014). *Clostridium difficile* binary toxin CDT: mechanism, epidemiology, and potential clinical importance. *Gut Microbes* 5 15–27. 10.4161/gmic.26854 24253566 PMC4049931

[B19] Goldenberg J. Z. Yap C. Lytvyn L. Lo C. K. Beardsley J. Mertz D. (2017). Probiotics for the prevention of *Clostridium difficile*-associated diarrhea in adults and children. *Cochrane Datab. Syst. Rev.* 12:CD006095. 10.1002/14651858.CD006095.pub4 PMC6486212 29257353

[B20] Hecht G. Pothoulakis C. Lamont J. T. Madara J. L. (1988). *Clostridium difficile* toxin A perturbs cytoskeletal structure and tight junction permeability of cultured human intestinal epithelial monolayers. *J. Clin. Invest.* 82 1516–1524. 10.1172/JCI113760 3141478 PMC442717

[B21] Hill C. Guarner F. Reid G. Gibson G. R. Merenstein D. J. Pot B. (2014). Expert consensus document. The international scientific association for probiotics and prebiotics consensus statement on the scope and appropriate use of the term probiotic. *Nat. Rev. Gastroenterol. Hepatol.* 11 506–514. 10.1038/nrgastro.2014.66 24912386

[B22] Hodges K. Gill R. (2010). Infectious diarrhea: cellular and molecular mechanisms. *Gut Microbes* 1 4–21. 10.4161/gmic.1.1.11036 21327112 PMC3035144

[B23] Imaoka A. Shima T. Kato K. Mizuno S. Uehara T. Matsumoto S. (2008). Anti-inflammatory activity of probiotic *Bifidobacterium*: enhancement of IL-10 production in peripheral blood mononuclear cells from ulcerative colitis patients and inhibition of IL-8 secretion in HT-29 cells. *World J. Gastroenterol.* 14 2511–2516. 10.3748/wjg.14.2511 18442197 PMC2708361

[B24] Jank T. Aktories K. (2008). Structure and mode of action of clostridial glucosylating toxins: the ABCD model. *Trends Microbiol.* 16 222–229. 10.1016/j.tim.2008.01.011 18394902

[B25] Kachrimanidou M. Malisiovas N. (2011). *Clostridium difficile* infection: a comprehensive review. *Crit. Rev. Microbiol.* 37 178–187. 21609252 10.3109/1040841X.2011.556598

[B26] Kachrimanidou M. Tsintarakis E. (2020). Insights into the role of human gut microbiota in *Clostridioides difficile* infection. *Microorganisms* 8:200. 10.3390/microorganisms8020200 PMC7074861 32023967

[B27] Kelly C. P. Pothoulakis C. Lamont J. T. (1994b). *Clostridium difficile* colitis. *N. Engl. J. Med.* 330 257–262. 8043060 10.1056/NEJM199401273300406

[B28] Kelly C. P. Keates S. Siegenberg D. Linevsky J. K. Pothoulakis C. Brady H. R. (1994a). IL-8 secretion and neutrophil activation by HT-29 colonic epithelial cells. *Am. J. Physiol.* 267 G991–G997. 7810667 10.1152/ajpgi.1994.267.6.G991

[B29] Kim J. J. Shajib M. S. Manocha M. M. Khan W. I. (2012). Investigating intestinal inflammation in DSS-induced model of IBD. *J. Vis. Exp.* 3678. 22331082 10.3791/3678 PMC3369627

[B30] Kim J. M. Kim J. S. Jun H. C. Oh Y. K. Song I. S. Kim C. Y. (2002). Differential expression and polarized secretion of CXC and CC chemokines by human intestinal epithelial cancer cell lines in response to *Clostridium difficile* toxin A. *Microbiol. Immunol.* 46 333–342. 10.1111/j.1348-0421.2002.tb02704.x 12139393

[B31] Kolling G. L. Wu M. Warren C. A. Durmaz E. Klaenhammer T. R. Timko M. P. (2012). Lactic acid production by *Streptococcus thermophilus* alters *Clostridium difficile* infection and *in vitro* toxin A production. *Gut Microbes* 3 523–529. 10.4161/gmic.21757 22895082 PMC3495789

[B32] Koon H. W. Su B. Xu C. Mussatto C. C. Tran D. H. Lee E. C. (2016). Probiotic *Saccharomyces boulardii* CNCM I-745 prevents outbreak-associated *Clostridium difficile*-associated cecal inflammation in hamsters. *Am. J. Physiol. Gastrointest. Liver Physiol.* 311 G610–G623. 10.1152/ajpgi.00150.2016 27514478 PMC5142203

[B33] Kuehne S. A. Cartman S. T. Minton N. P. (2011). Both, toxin A and toxin B, are important in *Clostridium difficile* infection. *Gut Microbes* 2 252–255. 10.4161/gmic.2.4.16109 21804353 PMC3260544

[B34] Le Bivic A. Hirn M. Reggio H. (1988). HT-29 cells are an *in vitro* model for the generation of cell polarity in epithelia during embryonic differentiation. *Proc. Natl. Acad. Sci. U.S.A.* 85 136–140. 10.1073/pnas.85.1.136 3277169 PMC279498

[B35] Lee Y. J. Arguello E. S. Jenq R. R. Littmann E. Kim G. J. Miller L. C. (2017). Protective factors in the intestinal microbiome against *Clostridium difficile* infection in recipients of allogeneic hematopoietic stem cell transplantation. *J. Infect. Dis.* 215 1117–1123. 10.1093/infdis/jix011 28498996 PMC5426375

[B36] Leelahavanichkul A. Panpetch W. Worasilchai N. Somparn P. Chancharoenthana W. Nilgate S. (2016). Evaluation of gastrointestinal leakage using serum (1–>3)-beta-D-glucan in a *Clostridium difficile* murine model. *FEMS Microbiol. Lett.* 363:fnw204. 10.1093/femsle/fnw204 27573235

[B37] Leffler D. A. Lamont J. T. (2015). *Clostridium difficile* infection. *N. Engl. J. Med.* 372 1539–1548. 25875259 10.1056/NEJMra1403772

[B38] Lemee L. Dhalluin A. Testelin S. Mattrat M. A. Maillard K. Lemeland J. F. (2004). Multiplex PCR targeting tpi (triose phosphate isomerase), tcdA (Toxin A), and tcdB (Toxin B) genes for toxigenic culture of *Clostridium difficile*. *J. Clin. Microbiol.* 42 5710–5714. 10.1128/JCM.42.12.5710-5714.2004 15583303 PMC535266

[B39] Li X. Chu Q. Huang Y. Xiao Y. Song L. Zhu S. (2019). Consortium of probiotics attenuates colonization of *Clostridioides difficile*. *Front. Microbiol.* 10:2871. 10.3389/fmicb.2019.02871 31921049 PMC6920126

[B40] Lyerly D. M. Krivan H. C. Wilkins T. D. (1988). *Clostridium difficile*: its disease and toxins. *Clin. Microbiol. Rev.* 1 1–18. 10.1128/CMR.1.1.1 3144429 PMC358025

[B41] Mack D. R. Michail S. Wei S. Mcdougall L. Hollingsworth M. A. (1999). Probiotics inhibit enteropathogenic *E. coli* adherence *in vitro* by inducing intestinal mucin gene expression. *Am. J. Physiol.* 276 G941–G950. 10198338 10.1152/ajpgi.1999.276.4.G941

[B42] Mooyottu S. Flock G. Upadhyay A. Upadhyaya I. Maas K. Venkitanarayanan K. (2017). Protective effect of Carvacrol against gut dysbiosis and *Clostridium difficile* associated disease in a mouse model. *Front. Microbiol.* 8:625. 10.3389/fmicb.2017.00625 28484429 PMC5399026

[B43] Mylonakis E. Ryan E. T. Calderwood S. B. (2001). *Clostridium difficile*–associated diarrhea: a review. *Arch. Intern. Med.* 161 525–533. 11252111 10.1001/archinte.161.4.525

[B44] Nusrat A. Von Eichel-Streiber C. Turner J. R. Verkade P. Madara J. L. Parkos C. A. (2001). *Clostridium difficile* toxins disrupt epithelial barrier function by altering membrane microdomain localization of tight junction proteins. *Infect. Immun.* 69 1329–1336. 10.1128/IAI.69.3.1329-1336.2001 11179295 PMC98024

[B45] Panpetch W. Chancharoenthana W. Bootdee K. Nilgate S. Finkelman M. Tumwasorn S. (2018). *Lactobacillus rhamnosus* L34 attenuates gut translocation-induced bacterial sepsis in murine models of leaky gut. *Infect. Immun.* 86 e700–e717. 10.1128/IAI.00700-17 29038123 PMC5736799

[B46] Panpetch W. Hiengrach P. Nilgate S. Tumwasorn S. Somboonna N. Wilantho A. (2020). Additional *Candida albicans* administration enhances the severity of dextran sulfate solution induced colitis mouse model through leaky gut-enhanced systemic inflammation and gut-dysbiosis but attenuated by *Lactobacillus rhamnosus* L34. *Gut Microbes* 11 465–480. 10.1080/19490976.2019.1662712 31530137 PMC7527076

[B47] Panpetch W. Somboonna N. Palasuk M. Hiengrach P. Finkelman M. Tumwasorn S. (2019). Oral *Candida* administration in a *Clostridium difficile* mouse model worsens disease severity but is attenuated by *Bifidobacterium*. *PLoS One* 14:e0210798. 10.1371/journal.pone.0210798 30645630 PMC6333342

[B48] Panpetch W. Spinler J. K. Versalovic J. Tumwasorn S. (2016). Characterization of *Lactobacillus salivarius* strains B37 and B60 capable of inhibiting IL-8 production in *Helicobacter pylori*-stimulated gastric epithelial cells. *BMC Microbiol.* 16:242. 10.1186/s12866-016-0861-x 27756217 PMC5070129

[B49] Paone P. Cani P. D. (2020). Mucus barrier, mucins and gut microbiota: the expected slimy partners? *Gut* 69 2232–2243. 10.1136/gutjnl-2020-322260 32917747 PMC7677487

[B50] Pfaffl M. W. (2001). A new mathematical model for relative quantification in real-time RT-PCR. *Nucleic Acids Res.* 29:e45. 10.1093/nar/29.9.e45 PMC55695 11328886

[B51] Pothoulakis C. (2000). Effects of *Clostridium difficile* toxins on epithelial cell barrier. *Ann. N. Y. Acad. Sci.* 915 347–356. 11193598 10.1111/j.1749-6632.2000.tb05263.x

[B52] Reeves A. E. Koenigsknecht M. J. Bergin I. L. Young V. B. (2012). Suppression of *Clostridium difficile* in the gastrointestinal tracts of germfree mice inoculated with a murine isolate from the family Lachnospiraceae. *Infect. Immun.* 80 3786–3794. 10.1128/IAI.00647-12 22890996 PMC3486043

[B53] Reeves A. E. Theriot C. M. Bergin I. L. Huffnagle G. B. Schloss P. D. Young V. B. (2011). The interplay between microbiome dynamics and pathogen dynamics in a murine model of *Clostridium difficile* infection. *Gut Microbes* 2 145–158. 10.4161/gmic.2.3.16333 21804357 PMC3225775

[B54] Reid G. Younes J. A. Van Der Mei H. C. Gloor G. B. Knight R. Busscher H. J. (2011). Microbiota restoration: natural and supplemented recovery of human microbial communities. *Nat. Rev. Microbiol.* 9 27–38. 10.1038/nrmicro2473 21113182

[B55] Ritchie M. L. Romanuk T. N. (2012). A meta-analysis of probiotic efficacy for gastrointestinal diseases. *PLoS One* 7:e34938. 10.1371/journal.pone.0034938 PMC3329544 22529959

[B56] Roychowdhury S. Cadnum J. Glueck B. Obrenovich M. Donskey C. Cresci G. A. M. (2018). *Faecalibacterium prausnitzii* and a prebiotic protect intestinal health in a mouse model of antibiotic and *Clostridium difficile* exposure. *JPEN J. Parenter. Enteral Nutr.* 42 1156–1167. 10.1002/jpen.1053 29385239 PMC6068000

[B57] Savidge T. C. Pan W. H. Newman P. O’brien M. Anton P. M. Pothoulakis C. (2003). *Clostridium difficile* toxin B is an inflammatory enterotoxin in human intestine. *Gastroenterology* 125 413–420. 10.1016/s0016-5085(03)00902-8 12891543

[B58] Schloss P. D. Westcott S. L. Ryabin T. Hall J. R. Hartmann M. Hollister E. B. (2009). Introducing mothur: open-source, platform-independent, community-supported software for describing and comparing microbial communities. *Appl. Environ. Microbiol.* 75 7537–7541. 10.1128/AEM.01541-09 19801464 PMC2786419

[B59] Shekels L. L. Lyftogt C. T. Ho S. B. (1996). Bile acid-induced alterations of mucin production in differentiated human colon cancer cell lines. *Int. J. Biochem. Cell Biol.* 28 193–201. 10.1016/1357-2725(95)00125-5 8729006

[B60] Shen N. T. Maw A. Tmanova L. L. Pino A. Ancy K. Crawford C. V. (2017). Timely use of probiotics in hospitalized adults prevents *Clostridium difficile* infection: a systematic review with meta-regression analysis. *Gastroenterology* 152 1889–1900.e1889. 10.1053/j.gastro.2017.02.003 28192108

[B61] Spinler J. K. Auchtung J. Brown A. Boonma P. Oezguen N. Ross C. L. (2017). Next-generation probiotics targeting *Clostridium difficile* through precursor-directed antimicrobial biosynthesis. *Infect. Immun.* 85 e303–e317. 10.1128/IAI.00303-17 28760934 PMC5607411

[B62] Sun X. Savidge T. Feng H. (2010). The enterotoxicity of *Clostridium difficile* toxins. *Toxins (Basel)* 2 1848–1880. 10.3390/toxins2071848 22069662 PMC3153265

[B63] Thomas C. M. Hong T. Van Pijkeren J. P. Hemarajata P. Trinh D. V. Hu W. (2012). Histamine derived from probiotic *Lactobacillus reuteri* suppresses TNF via modulation of PKA and ERK signaling. *PLoS One* 7:e31951. 10.1371/journal.pone.0031951 22384111 PMC3285189

[B64] Thomas C. M. Saulnier D. M. Spinler J. K. Hemarajata P. Gao C. Jones S. E. (2016). FolC2-mediated folate metabolism contributes to suppression of inflammation by probiotic *Lactobacillus reuteri*. *Microbiologyopen* 5 802–818. 10.1002/mbo3.371 27353144 PMC5061717

[B65] Trejo F. M. Perez P. F. De Antoni G. L. (2010). Co-culture with potentially probiotic microorganisms antagonises virulence factors of *Clostridium difficile in vitro*. *Antonie Van Leeuwenhoek* 98 19–29. 10.1007/s10482-010-9424-6 20232250

[B66] Velcich A. Augenlicht L. H. (1993). Regulated expression of an intestinal mucin gene in HT29 colonic carcinoma cells. *J. Biol. Chem.* 268 13956–13961. 7686147

[B67] Viswanathan V. K. Hodges K. Hecht G. (2009). Enteric infection meets intestinal function: how bacterial pathogens cause diarrhoea. *Nat. Rev. Microbiol.* 7 110–119. 19116615 10.1038/nrmicro2053 PMC3326399

[B68] Voth D. E. Ballard J. D. (2005). *Clostridium difficile* toxins: mechanism of action and role in disease. *Clin. Microbiol. Rev.* 18 247–263. 10.1128/CMR.18.2.247-263.2005 15831824 PMC1082799

[B69] Wei Y. Yang F. Wu Q. Gao J. Liu W. Liu C. (2018). Protective effects of bifidobacterial strains against toxigenic *Clostridium difficile*. *Front. Microbiol.* 9:888. 10.3389/fmicb.2018.00888 29867801 PMC5952185

[B70] Xu Q. Gu S. Chen Y. Quan J. Lv L. Chen D. (2018). Protective effect of *Pediococcus pentosaceus* LI05 against *Clostridium difficile* infection in a mouse model. *Front. Microbiol.* 9:2396. 10.3389/fmicb.2018.02396 PMC6189400 30356740

[B71] Xue Y. Zhang H. Wang H. Hu J. Du M. Zhu M. J. (2014). Host inflammatory response inhibits *Escherichia coli* O157:H7 adhesion to gut epithelium through augmentation of mucin expression. *Infect. Immun.* 82 1921–1930. 10.1128/IAI.01589-13 24566630 PMC3993425

[B72] Yong C. C. Lim J. Kim B. K. Park D. J. Oh S. (2019). Suppressive effect of *Lactobacillus fermentum* Lim2 on *Clostridioides difficile* 027 toxin production. *Lett. Appl. Microbiol.* 68 386–393. 10.1111/lam.13124 30714187

[B73] Zemljic M. Rupnik M. Scarpa M. Anderluh G. Palu G. Castagliuolo I. (2010). Repetitive domain of *Clostridium difficile* toxin B exhibits cytotoxic effects on human intestinal epithelial cells and decreases epithelial barrier function. *Anaerobe* 16 527–532. 10.1016/j.anaerobe.2010.06.010 20620216

