# Supplementary figures and images for: Lacticaseibacillus casei Strain T21 Attenuates Clostridioides difficile Infection in a Murine Model Through Reduction of Inflammation and Gut Dysbiosis With Decreased Toxin Lethality and Enhanced Mucin Production
Source: Front Microbiol. 2021 Dec 1;12:745299. doi: 10.3389/fmicb.2021.745299 (PMC8672038; doi:10.3389/fmicb.2021.745299)

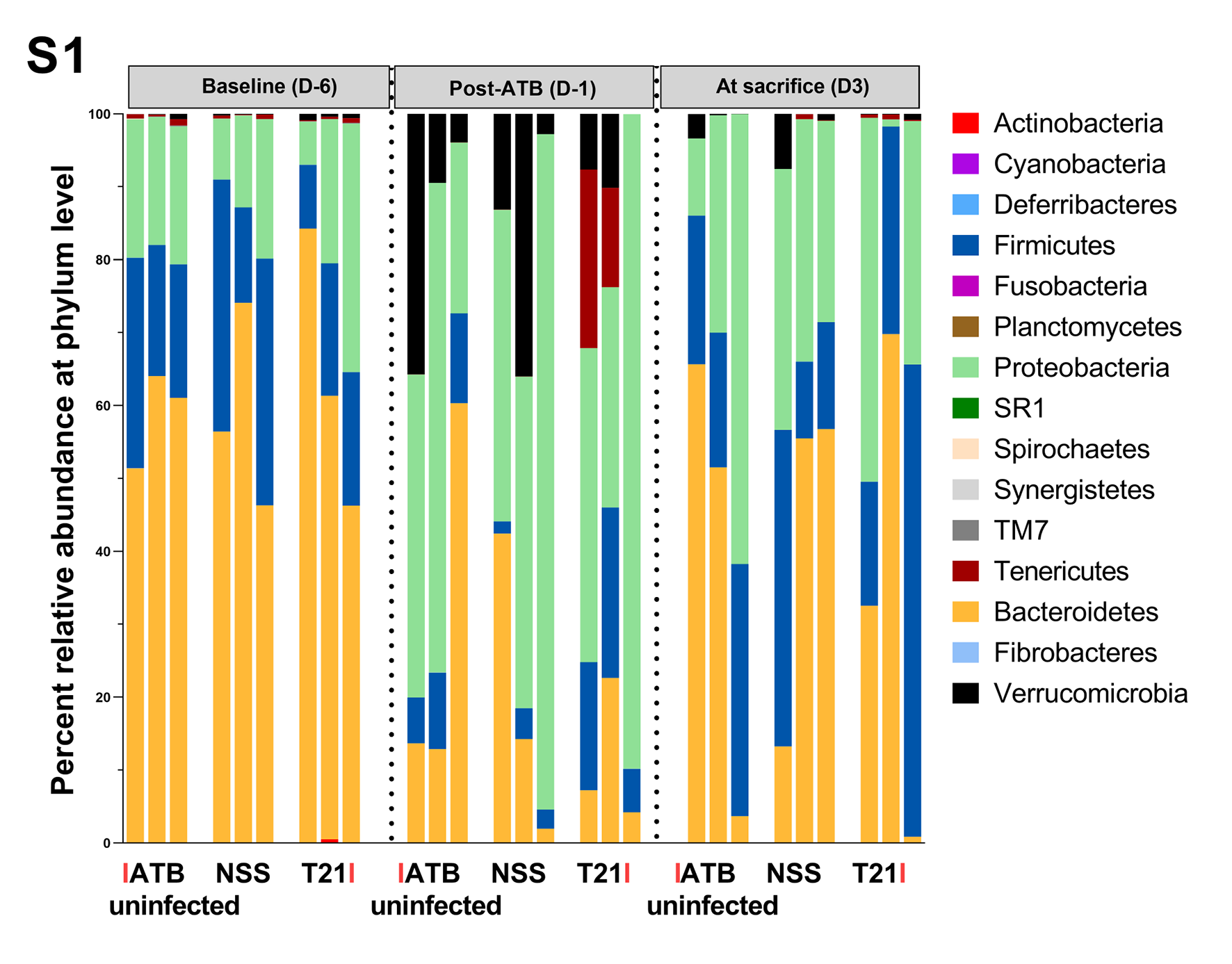

Supplement: Supplementary file 1 [file Image_1.TIF]

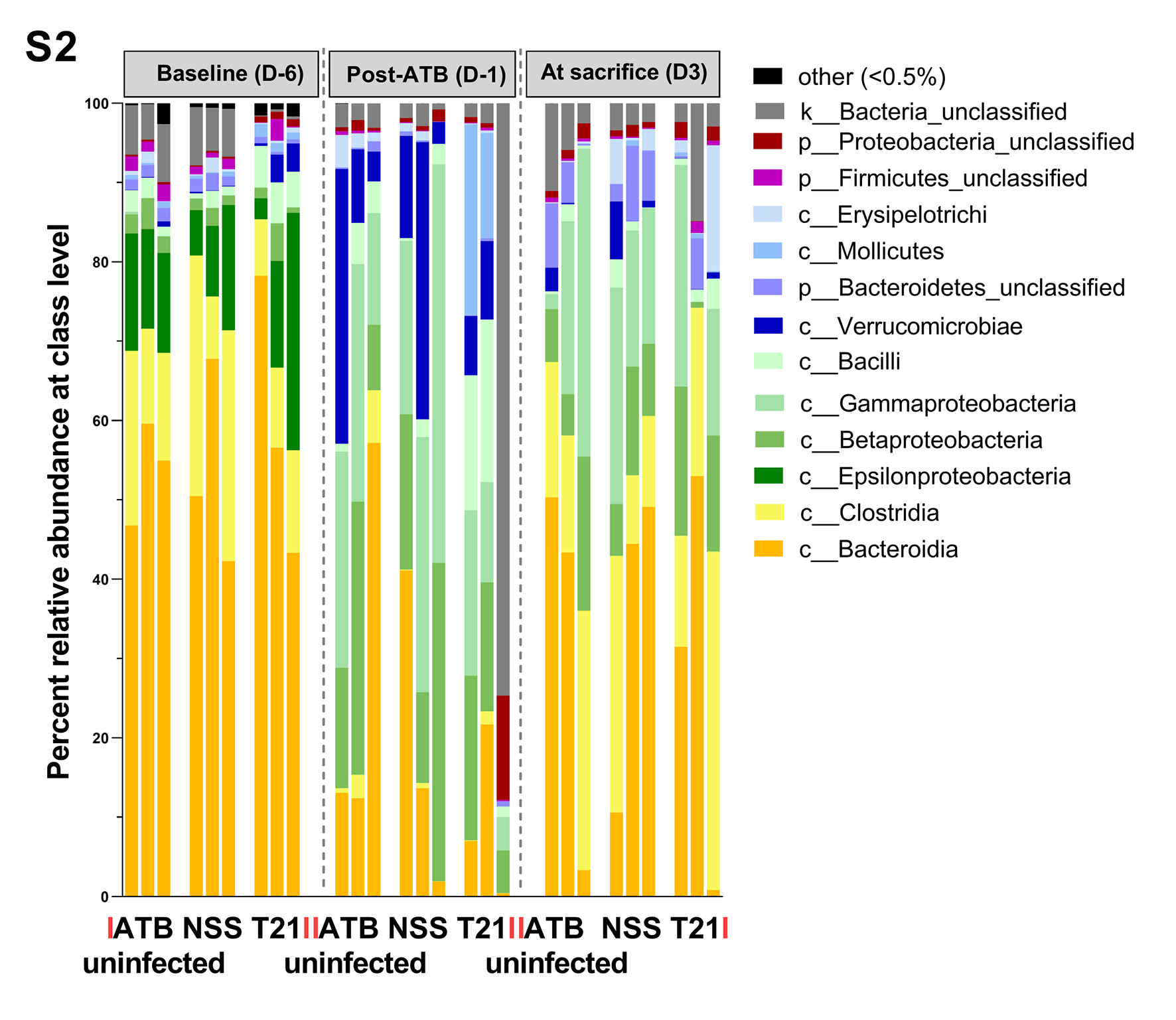

Supplement: Supplementary file 2 [file Image_2.TIF]

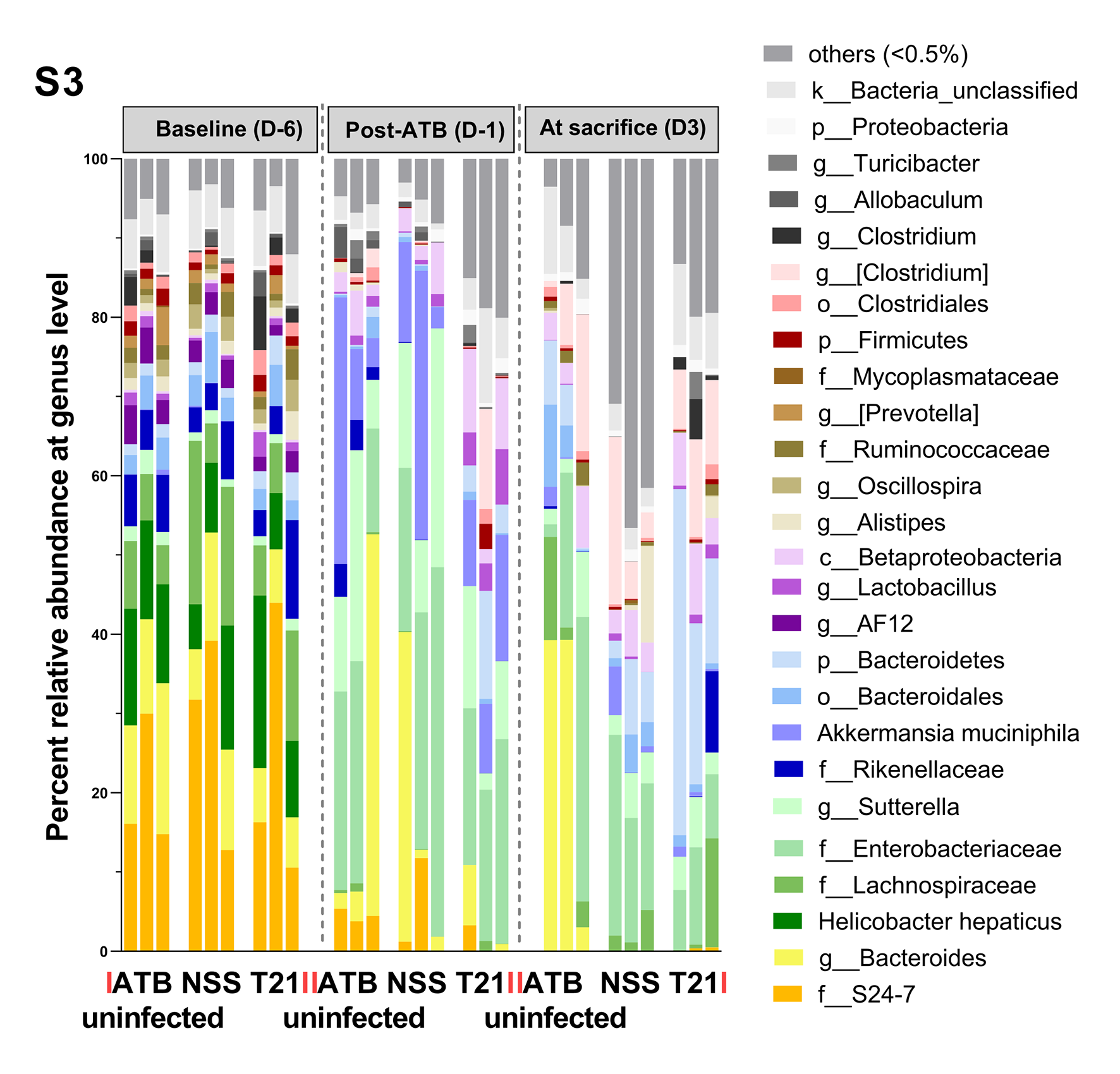

Supplement: Supplementary file 3 [file Image_3.TIF]
